# Role of Janus Kinase (JAK) Inhibitor in Autoimmune Ocular Inflammation: A Systematic Review

**DOI:** 10.1155/2021/2324400

**Published:** 2021-12-20

**Authors:** Ji Wen, Huifang Hu, Menglin Chen, Hang Yang, Yi Zhao, Yi Liu

**Affiliations:** ^1^Department of Rheumatology and Immunology, West China Hospital, Sichuan University, Chengdu, Sichuan, China; ^2^Department of Occupational Health and Toxicity/Nephrology, West China Fourth Hospital, Sichuan University, Chengdu, Sichuan, China

## Abstract

**Purpose:**

To evaluate the effectiveness of Janus kinase (JAK) inhibitors for the treatment of patients with autoimmune disease and associated inflammatory ocular diseases.

**Methods:**

We identified relevant literature by screening the MEDLINE, PubMed, and Cochrane databases for randomized controlled trials, cohort studies, case controls, and case reports.

**Results:**

Seven studies, including 11 patients, were included in the final systematic analysis. Of the 11 patients, there were 5 cases of juvenile idiopathic arthritis- (JIA-) associated uveitis, 1 case of rheumatoid arthritis- (RA-) associated keratitis, 1 case of RA-associated scleritis, 1 case of psoriasis-associated conjunctivitis, 2 cases of noninfectious scleritis, and 1 case of uveitis with suspected autoimmune disease. None of these 11 patients responded adequately to conventional treatments, including biological agents; these were all refractory cases and switched to JAK inhibitor therapy. Irrespective of whether they were suffering from uveitis, scleritis, or other types of ocular inflammation, all 11 patients showed an improvement to JAK inhibitors without significant side effects. Different types of JAK inhibitors might be associated with different responses when used to treat ocular inflammation.

**Conclusions:**

JAK inhibitors may represent an alternative treatment option for patients with autoimmune ocular inflammation.

## 1. Introduction

Noninfectious inflammatory ocular diseases can occur in isolation or in the context of systemic autoimmune diseases, such as rheumatoid arthritis (RA), juvenile idiopathic arthritis (JIA), ankylosing spondylitis (AS), and systemic vasculitis (SV). Ocular inflammation includes a diverse group of ocular inflammatory diseases that frequently present in the form of scleritis, keratitis, uveitis, conjunctivitis, and retinitis; these conditions can lead to a number of other vision-threatening ocular complications.

Currently, the traditional treatments for such ocular complications are nonsteroidal anti-inflammatory drugs (NSAIDs), corticosteroids, and conventional disease-modifying antirheumatic drugs (cDMARDs) [1]. However, some patients are nonresponsive to such therapies. Several classes of biological agents have been reported to control ocular inflammation, including TNF-alpha blockers, tocilizumab, and rituximab [2–5]. However, the literature also reports that some severe cases were refractory and failed to reach remission [2, 3].

The Janus kinase (JAK) pathway plays a key role in inflammatory cell regulation, cytokine production, and proinflammatory signal transduction [6, 7]. Dysregulation of the JAK pathway is associated with the pathogenesis of various inflammatory and autoimmune disorders. Therefore, JAK inhibitors have the potential to alleviate the inflammatory process. However, the applications of JAK inhibitors are relatively new in terms of clinical therapy, particularly for autoimmune diseases. Study data is not abundantly available for this particularly field-of-interest. In this study, we aimed to summarize and analyze existing evidence related to the efficacy of different JAK inhibitors with regard to controlling ocular inflammation.

## 2. Methods

### 2.1. Inclusion and Exclusion Criteria

We conducted a retrospective and systematic evaluation of patients with noninfectious inflammatory ocular diseases who were treated with JAK inhibitors. We examined a range of literature types, including randomized controlled trials, cohort studies, and case reports. These articles involved a range of inflammatory ocular diseases, including uveitis, scleritis, keratitis, conjunctivitis, and retinitis. During our literature searches, we defined JAK inhibitors as tofacitinib, baricitinib, jakinib, ruxolitinib, and filgotinib.

Articles were excluded if any infectious pathogen was involved. We also excluded research involving animal experiments and literature that had been duplicated, was incomplete, or contained obvious errors.

### 2.2. Search Strategy

Literature searches were carried out by two independent investigators. The investigators screened the MEDLINE, PubMed, and Cochrane databases for relevant articles that were published from inception to March 2021. The search algorithm included several keywords connected by Boolean operator reported to control ocular inflammation. First, the keywords “Janus Kinase inhibitor”, “JAK inhibitor”, “tofacitinib”, “baricitinib”, “jakinib”, “ruxolitinib” and “filgotinib” were connected by the Boolean operator “OR”. Next, the keywords “ocular inflammation”, “episcleritis”, “scleritis”, “uveitis”, “keratitis”, “conjunctivitis”, “retinal vasculitis”, and “retinitis” were connected by the Boolean operator “OR”. Finally, these search results were connected by the Boolean operator “AND”. Two investigators independently searched and assessed the published studies. Any disagreement was resolved by consensus.

### 2.3. Statistical Analysis

We retrospectively collated a range of demographic, clinical, and therapeutic data, including authors, publication date, country of origin, gender, age, disease duration, diagnoses, complications, previous therapy history, and treatment outcomes. Data were analyzed using IBM SPSS Statistics for Windows, version 24. For quantitative variables, we calculated the mean and standard deviation.

## 3. Results

### 3.1. Study Selection and Features

**Figure 1:**
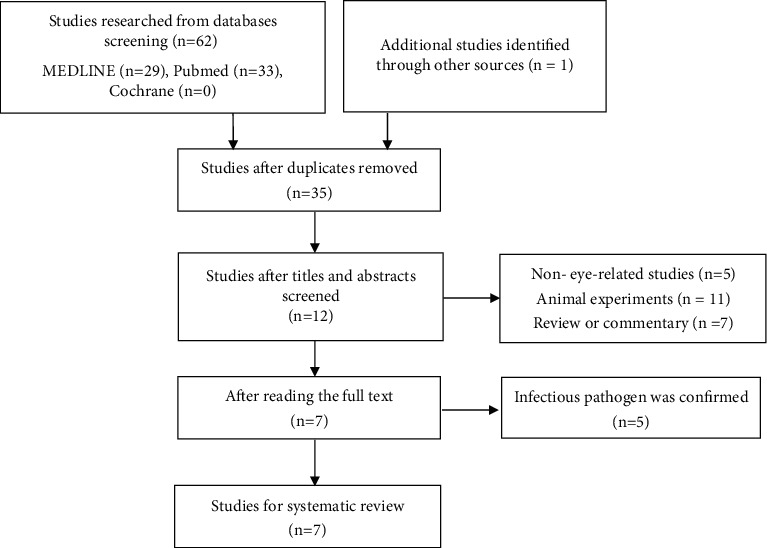
The flow diagram of the reviewed literature.

A total of 63 articles were separately identified from MEDLINE, PubMed, and Cochrane databases.  Figure 1 provides flow diagram showing the process used to review the literature. All the identified articles were case reports; our literature searches did not identify any relevant randomized controlled trials, cohort studies, or cross-sectional surveys. The earliest case report was published in 2014. A total of 7 articles reported the therapeutic effects of JAK inhibitors when used to treat ocular inflammation; 11 patients were included [8–14]. The basic features of all included articles are summarized in Table 1.

### 3.2. Demographic and Clinical Features of Patients

We identified 11 patients who previously presented with ocular inflammation and received therapeutic management involving JAK inhibitors. Of the 11 patients, 5 (45.45%) had JIA-associated uveitis [11, 12], 1 (9.09%) had RA-associated keratitis [8], 1 (9.09%) had RA-associated scleritis [14], 1 (9.09%) had psoriasis-associated conjunctivitis [9], 2 (18.18%) had noninfectious scleritis, and 1 (9.09%) had uveitis with suspected autoimmune disease [10, 13] (Table 1). According to the classification proposed by the Standardization of Uveitis Nomenclature (SUN) Working Group [15], there were 6 patients with uveitis including 2 cases of anterior uveitis (33.33%), 3 cases of panuveitis (50.00%), and 1 case of anterior uveitis and intermediate uveitis (16.67%). These patients suffered from the abovementioned inflammations of ocular tissue and even had complications including macular edema, retinal detachment, cataract, band keratopathy, and glaucoma. In these articles, there were 3 males and 8 females whose mean age and mean disease duration were, respectively, 39.82 ± 14.94 years (range: 18-65 years) and 16.13 ± 12.18 years (range: 2-34 years) (Table 2).

### 3.3. Previous Therapeutic Histories

Some of the identified patients received therapies involving conventional DMARDs. All patients had a long-term history of ocular inflammation, received complicated therapies, and were unable to achieve a long-term and stable resolution. Monotherapy involving conventional DMARDs was commonly reported to be ineffective. Even in combined therapeutic approaches, most of the identified patients failed to show adequate improvement; some even presented with obvious side effects (Table 3).

Our literature search revealed that biological inhibitors only provided temporary relief (Table 4). These patients experienced frequent flares of systemic symptoms and ocular symptoms. Most of the patients were treated with combined therapeutic approach involving multiple forms of steroids including 6 patients treated with prednisone, 1 patient treated with methylprednisolone, and 1 patient treated with dexamethasone. Three of them completely received the local injection and the topical and oral administration of steroids; six of them were treated in one or two ways. Ocular inflammations were refractory to topical steroid drops. Local steroid injections often led to transient relief. Oral prednisone was often effective but was difficult to taper without inducing flares (Table 5).

Due to serious complications, some of the patients underwent surgery. Patient number 1 underwent a corneal gluing procedure of the right eye [8]. Patient number 2 received bilateral implantation of fluocinolone acetonide intravitreal implants [10]. Patient number 4 received cataract surgery with intraocular lens (IOL) implantation in both eyes and vitrectomy in her right eye [11]. Patient number 6 underwent cataract extraction [12]. Finally, patient number 9 underwent cataract extraction with IOL implantation [12].

### 3.4. JAK Inhibitor Treatment

No matter which type of JAK inhibitor was used, all of the case reports, except for patient number 8 [12], showed good efficacy with regard to ocular symptoms. Irrespective of whether a patient received monotherapy or combined treatment, almost all gained some form of control over their condition. With regard to systemic symptoms, the combination of baricitinib with MTX and prednisone still showed an incomplete treatment response with relapsing episodes of active joint inflammation. While taking tofacitinib, none of the reported systemic symptoms were active. The available literature suggests that it might be easier to control systemic symptoms. In some case reports, the response to tofacitinib treatment was rapid; inflammation was usually resolved within one or two weeks (Table 6).

Literature analysis showed that side effects were rare. Only patient number 11 experienced a low level of neutrophil granulocytes during treatment involving baricitinib and MTX [9]. This led to the discontinuation of baricitinib for 2 weeks; subsequently, the patient did not experience any adverse effects. However, we cannot rule out the potential adverse effects of MTX (Table 6).

Some of the literature described the use of JAK inhibitors to treat patients with uveitis and scleritis; the grading of the anterior chamber cells decreased from pretreatment to posttreatment. Other ocular indicators, including best corrected visual acuity and central foveal thickness, showed obvious improvements (Table 7).

## 4. Discussion

The eyeball is composed of different layers and has a separated immune environment. Multiple mechanisms contribute to local immune tolerance, including the absence of vessels in the cornea and the anterior chamber, immunosuppressive factors, and inflammatory regulation via the anterior chamber [16]. The posterior segment of the eye is also a unique structure that contains photoreceptor cells and retinal pigment epithelium; this forms a physical barrier that separates the systemic immune system from the retinal space [17]. However, inflammatory rheumatic diseases can affect multilayer structures and have destructive effects on the ocular microenvironment. Therefore, inflammatory ophthalmic disorders are a group of heterogeneous inflammatory conditions that affect different anatomical ocular tissues, involving scleritis, keratitis, anterior uveitis, posterior uveitis, and retinal vasculitis; these occur in isolation or in the context of systemic autoimmune diseases. Systemic autoimmune diseases that include ocular involvement are also a group of diverse diseases, including rheumatoid arthritis, juvenile idiopathic arthritis, systemic vasculitis, systemic lupus erythematosus, Behçet's syndrome, and relapsing polychondritis [18].

In this article, we reviewed 5 cases of JIA-associated uveitis, 1 case of RA-associated keratitis, 1 case of RA-associated scleritis, 1 case of psoriasis-associated conjunctivitis, 2 cases of noninfectious scleritis, and 1 case of uveitis with suspected autoimmune disease.

Our results are consistent with other reports. JIA-associated uveitis is the most common rheumatic ocular involvement in pediatric patients [19]. The estimated prevalence of uveitis in patients with JIA ranges from 11.6% [20] to 30% [21]. The most common form was chronic anterior uveitis, as defined by the classification scheme published by the Standardization of Uveitis Nomenclature (SUN) Working Group [15, 22]. In a retrospective review, 68.3% of 1081 JIA cases were shown to have chronic anterior uveitis [23]. Acute anterior uveitis accounted for 16.2%, recurrent anterior uveitis reached 12%, and panuveitis was just 3.5% [23]. Of the multiple etiological factors responsible for noninfectious scleritis, RA represents a major cause [24]. The anterior segment is more commonly affected than the posterior segment in RA-related ocular complications. In a previous study of 243 patients with scleritis, the most frequent rheumatic disease was RA (15.2%) [25]. In other studies, RA-related scleritis accounted for approximately 25% of all cases [26]. RA-related keratitis is also common in patients with active scleritis [18]. However, compared with spondyloarthritis, RA is a rare cause of uveitis [27]. Ophthalmic manifestations are estimated to occur in 10% of patients with psoriasis and 31% of patients with psoriatic arthritis (PsA) [28]. Another study reported that the leading ocular disorder in PsA patients was conjunctivitis (19.6%), followed by iritis (7.1%) [29].

Our review of the literature revealed that most of these cases received treatments that included glucocorticoids, several conventional disease-modifying antirheumatic drugs, and multiple biological agents. However, patients did not show adequate improvements or achieve long-term resolutions; they even presented with obvious side effects and faced dilemmas as to whether to continue treatment or not. The statuses of the ocular and systematic inflammation in most of the patients reviewed were refractory and severe.

A range of JAK inhibitors have been or are being developed, for the treatment of refractory cases and those with various autoimmune diseases, including rheumatoid arthritis, psoriatic arthritis, ulcerative colitis, and ankylosing spondylitis [30–32]. Many of the cytokines involved in autoimmune and inflammatory diseases utilize JAKs and STATs to transduce intracellular signals. JAK inhibitors are less selective than biological inhibitors, can simultaneously block the signaling of multiple cytokine axis, and offer new therapeutic strategies [33]. Whether these inhibitors could simultaneously have therapeutic effects on ocular complications remains unclear. Unfortunately, the review of the literature failed to identify publications involving a large case series or randomized controlled trials. We only identified several case reports that indicated the anti-inflammatory effects of JAK inhibitors on the inflammation caused in a diverse range of ocular tissues by different rheumatic diseases.

It is important that we consider why JAK inhibitors exhibit the potential to play a role in autoimmune-related ocular inflammatory diseases. Dysregulation of the JAK-STAT pathway is known to be associated with the pathogenesis of various inflammatory and autoimmune disorders [33, 34]. The JAK-STAT pathway is known to be important for inflammatory cell regulation, cytokine production, and proinflammatory signal transduction [6, 7].

Although the etiology of ocular inflammation has yet to be fully elucidated, it is possible that the JAK/STAT pathway may participate in ocular pathology because this mechanism regulates the differentiation of pathogenic Th1 and Th17 cells. The Th1 and Th17 cell subsets require STAT1 and STAT3 during development and may be the etiological agents responsible for human uveitis and scleritis and experimental autoimmune uveoretinitis [35–37]. In the mouse model of uveitis, inhibition of the JAK/STAT signaling pathway by SOCS1-KIR, which binds to JAK2, could suppress and ameliorate experimental autoimmune uveitis (EAU) [38]. The mechanism that is responsible for this action involves downregulating the proliferation of pathogenic Th17 cells and inhibiting the migration of inflammatory cells into the neuroretina during EAU. However, some researchers have reported that the effect of tofacitinib on Th1/Th17 balance in the EAU model was different from the effects induced by SOCS1-KIR. Tofacitinib inhibited the development of EAU by reducing the proportion of Th1 cells instead of Th17 cells, and by suppressing the production of IFN-*γ*, did not exert effect on the expression of IL-17 and its transcription factor ROR*γ*t [39]. JAK inhibitors can control both intraocular inflammation and ocular surface inflammation. In an animal model of experimental dry eye, the application of a topical JAK inhibitor (tofacitinib) suppressed ocular surface inflammation and immunity in an experimental model of corneal thermocautery [40]. Even in the conjunctive structure of ocular tissue, tofacitinib has also been shown to prevent experimental allergic conjunctivitis in BALB/c mice by downregulating the phosphorylation of JAK3/STAT signaling [41].

This retrospective review had some limitations that need to be considered, including the lack of a control group, the small number of patients, and the lack of high-level evidence-based studies. However, we believe that all of these cases reported herein are valuable and can facilitate the future direction of our research. Future research may prove that JAK inhibitors can provide a novel treatment option for refractory autoimmune-related ocular inflammation.

## 5. Conclusion

JAK inhibitors may represent an alternative treatment option for patients with autoimmune-related ocular inflammation.

## Figures and Tables

**Table 1:** Features of the case reports included in the analysis.

Author	Year	Country	Type	Number	Systemic disease	Eye involvement	Intervation
Philip B Meadow [8]	2014	America	Case report	1	RA	Keratitis	Tofacitinib
Stephanie Sarny [9]	2018	Austria	Case report	1	Psoriasis, MMP	Conjunctivitis	Baricitinib
Michael A. Paley [10]	2019	America	Case report	2	NA	Uveitis scleritis	Tofacitinib
P. Bauermann [11]	2019	Germany	Case report	1	JIA	Uveitis	Tofacitinib
Elisabetta Miserocchi [12]	2020	Italy	Case series	4	JIA	Uveitis	Baricitinib Tofacitinib
Richa Pyare [13]	2020	India	Case report	1	NA	Scleritis	Tofacitinib
Claudia Fabiani [14]	2020	Italy	Case report	1	RA	Scleritis	Tofacitinib

JIA: juvenile idiopathic arthritis; RA: rheumatoid arthritis; MMP: mucous membrane pemphigoid; PsA: psoriatic arthritis; NA: not available.

**Table 2:** Demographic and clinical features of patients.

Patient	Author	Gender	Age	Ocular inflammation	Detail of ocluar inflammation	Systematic disease	Disease duration
1	Philip B. meadow	Female	59	Keratitis	Unilateral ulcerative keratitis (right eye), injection of the conjunctiva, pericentral ulceration of the cornea,stromal thinning, pannus, punctate epithelial erosion	RA	9 years
2	Michael A.	Female	45	Anterior and intermediate uveitis	Bilateral anterior uveitis with hypopyon, Vitritis, cystoid macular edema	Undefined	NA
3	Michael A.	Female	40	Scleritis	Bilateral scleritis	NA	NA
4	P. Bauermann	Female	22	Anterior uveitis	Bilateral anterior uveitis with macular edema	JIA(oligo-extended)	20years
5	Claudia Fabiani	Female	45	Scleritis	Bilateral anterior scleritis	RA	NA
6	Elisabetta Miserocchi	Female	43	Panuveitis	Bilateral aggressive anterior uveitis; cataract, band keratopathy, macular edema and retinal vasculitis, retinal detachment and phthisis bulbi; finally bilateral, chronic panuveitis	JIA(oligo-extended)	33year
7	Elisabetta Miserocchi	Female	18	Panuveitis	Bilateral anterior uveitis at first; bilateral chronic panuveitis during follow-up cataract, band keratopathy, glaucoma	JIA(polyarticular)	17years
8	Elisabetta Miserocchi	Female	37	Anterior uveitis	Bilateral anterior uveitis, cataract, band keratopathy	JIA(oligo-extended)	34years
9	Elisabetta Miserocchi	Male	21	Panuveitis	Unilateral anterior uveitis(right eye), chronic panuveitis cataract, band keratopathy, macular edema	JIA(polyarticular)	6years
10	Richa Pyare	Male	65	Scleritis	Deep episcleral congestion, active necrotizing scleritis with immature senile cataract	NA	2years
11	Stephanie Sarny	Male	43	Conjunctivitis	Bilateral conjunctivitis, subconjunctival fibrosis, symblepharon, corneal neovascularization	Psoriasis, mucous membrane pemphigoid	8years

Note: NA: not available.

**Table 3:** Previous therapy history of conventional DMARDs.

Patient	Gender	Age	Ocular inflammation	MTX	CTX	CsA	MMF	LEF	AZA
1	Female	59	Keratitis	+	NA	NA	NA	NA	NA
2	Female	45	Anterior and intermediate uveitis	+	NA	NA	+	+	+
3	Female	40	Scleritis	+	+	NA	+	NA	+
4	Female	22	Anterior uveitis	+	NA	+	+	NA	NA
5	Female	45	Scleritis	NA	NA	NA	NA	NA	NA
6	Female	43	Panuveitis	NA	NA	NA	NA	+	NA
7	Female	18	Panuveitis	+	NA	NA	NA	NA	NA
8	Female	37	Anterior uveitis	+	NA	NA	NA	NA	+
9	Male	21	Panuveitis	+	NA	+	NA	NA	NA
10	Male	65	Scleritis	NA	NA	NA	+	NA	NA
11	Male	43	Conjunctivitis	+	+	NA	+	NA	NA

Notes: MTX: methotrexate; CTX: cyclophosphamide; CsA: cyclosporine A; MMF: mycophenolate mofetil; LEF: leflunomide; AZA: azathioprine; NA: not available.

**Table 4:** Previous therapy history of biological DMARDs.

Patient	Gender	Age	Ocular inflammation	ADA	IFX	ETN	RTX	GOL	CER	ABA	TCZ
1	Female	59	Keratitis	NA	NA	NA	NA	NA	NA	+	NA
2	Female	45	Anterior and intermediate uveitis	+	+	NA	NA	NA	+	NA	NA
3	Female	40	Scleritis	NA	NA	NA	NA	NA	NA	NA	NA
4	Female	22	Anterior uveitis	+	+	NA	+	+	NA	NA	+
5	Female	45	Scleritis	+	NA	+	+	NA	NA	NA	+
6	Female	43	Panuveitis	+	+	NA	+	NA	NA	+	+
7	Female	18	Panuveitis	+	+	NA	+	NA	NA	+	NA
8	Female	37	Anterior uveitis	+	+	NA	NA	+	NA	NA	+
9	Male	21	Panuveitis	+	+	+	+	NA	NA	+	+
10	Male	65	Scleritis	NA	NA	NA	NA	NA	NA	NA	NA
11	Male	43	Conjunctivitis	+	NA	NA	+	NA	NA	NA	NA

Notes: ADA: adalimumab; IFX: infliximab; ETN: etanercept; RTX: rituximab; GOL: golimumab; CER, certolizumab pegol; ABA: abatacept; TCZ: tocilizumab; NA: not available.

**Table 5:** Previous therapy history of corticosteroids.

Patient	Gender	Age	Ocular inflammation	Corticosteroid	Dosage	Topical	Local Injection	Oral
1	Female	59	Keratitis	Methylprednisolone Prednisoneacetate	Prednisoneacetate 1% 1 drop tid	+	NA	NA
2	Female	45	Anterior and intermediate uveitis	Prednisone	80 mg bid	+	+	+
3	Female	40	Scleritis	Prednisone	12 mg qd	+	+	+
4	Female	22	Anterior uveitis	Dexamethasone	700ug	NA	+	NA
5	Female	45	Scleritis	NA	NA	NA	NA	NA
6	Female	43	Panuveitis	NA	NA	NA	NA	NA
7	Female	18	Panuveitis	Prednisone	12.5 mg qd	+	+	+
8	Female	37	Anterior uveitis	NA	NA	NA	+	NA
9	Male	21	Panuveitis	Steroids	NA	NA	+	+
10	Male	65	Scleritis	Prednisolone	1 mg/kg qd	+	NA	+
11	Male	43	Conjunctivitis	Prednisone	NA	NA	NA	+

Note: NA: not available.

**Table 6:** Characteristics of JAK inhibitor treatment.

Patient	Gender	Age	Ocular inflammation	Inhibitor	Dosage	Treatment duration	Combined therapy	Side effects	Systematic symptoms	Ocular symptoms
1	Female	59	Keratitis	Tofacitinib	5 mg bid	1 month; Improved after 2 weeks	NA	No	Inactive	Inactive
2	Female	45	Anterior and intermediate uveitis	Tofacitinib	11 mg daily	4months; Improved after 1 month	MTX	No	Combination: Inactive Monotherapy: Active	Inactive
3	Female	40	Scleritis	Tofacitinib	11 mg daily	9 months; Improved after 1 week	MTX	No	No systematic symptoms	Inactive
4	Female	22	Anterior uveitis	Tofacitinib	5 mg bid	9 months	MTX 2.5 mg qod	No	NA	Inactive
5	Female	45	Scleritis	Tofacitinib	5 mg bid	6 months	Prednisone 5 mg qd	No	NA	Inactive
6	Female	43	Panuveitis	Tofacitinib	5 mg bid	7 months	NA	No	Inactive	Inactive
7	Female	18	Panuveitis	Baricitinib	4 mg Qd	5 months	MTX 15 mg qw Prednisone 12.5 mg qd	No	Active	Inactive
8	Female	37	Anterior uveitis	Baricitinib	4 mg qd	13 months	NA	No	Inactive	Active
9	Male	21	Panuveitis	Baricitinib	4 mg qd	4 months	MTX 15 mg qw Prednisone 7.5 mg qd	No	Active	Inactive
10	Male	65	Scleritis	Tofacitinib	5 mg bid	Improvd after 1 month	MMF 500 mg bid Prednisone 2.5 mg qod	No	No systematic symptoms	Inactive
11	Male	43	Conjunctivitis	Baricitinib	4 mg qd	6month; Improvd after 2 weeks	MTX 25 mg qw Prednisolone 6 mg qd	Yes	NA	Inactive

Note: NA: not available.

**Table 7:** Treatment response of ocular inflammation.

Patient	Gender	Age	Ocular inflammation	BCVA	ACC	CFT
1	Female	59	Keratitis	Pre:RE:20/200 LE:20/20 Post:RE:20/30	Pre:RE0	NA
2	Female	45	Anterior and intermediate uveitis	NA	Pre:RE2+ LE2+ Post: RE0.5+ LE0	NA
3	Female	40	Scleritis	NA	NA	NA
4	Female	22	Anterior uveitis	Pre:RE20/100 LE20/200 Post:RE20/25 LE20/32	Pre:RE3+ LE0+ Post:RE0 LE0	Pre: RE468 LE630 Post: RE252 LE254
5	Female	45	Scleritis	NA	NA	NA
6	Female	43	Panuveitis	Pre:RE:20/40 LE:No light perception	Pre:2+ Post:0	Pre:350 Post:270
7	Female	18	Panuveitis	Post:RE:20/40 LE:20/200	Pre:3+ Post:0.5+	Pre:320 Post:264
8	Female	37	Anterior uveitis	Post:RE: 20/60 LE: 20/60	Pre:2+ Post:0	Pre:450 Post:276
9	Male	21	Panuveitis	Post:RE: 20/20 LE: 20/20	Pre:3+ Post:0.5+	Pre:400 Post:280
10	Male	65	Scleritis	Pre:RE6/ 6 LE 6/36 Post:LE6/24	Pre:1+ Post:0	NA
11	Male	43	Conjunctivitis	Pre:RE20/30 LE:Counting fingers	NA	NA

Note: ACC: anterior chamber cell; BCVA: best corrected visual acuity; CFT: central foveal thickness: Pre: pretreatment; Post: posttreatment; RE: right eye; LE: left eye; NA: not available.
